# Temporally modulated pulsed proton re-irradiation (TMPPR) for recurrent high-grade gliomas

**DOI:** 10.1093/noajnl/vdad074

**Published:** 2023-06-15

**Authors:** Alonso La Rosa, Alonso N Gutierrez, Yazmin Odia, Michael W McDermott, Manmeet S Ahluwalia, Minesh P Mehta, Rupesh Kotecha

**Affiliations:** Department of Radiation Oncology, Miami Cancer Institute, Baptist Health South Florida, Miami, FL, USA; Department of Radiation Oncology, Miami Cancer Institute, Baptist Health South Florida, Miami, FL, USA; Herbert Wertheim College of Medicine, Florida International University, Miami, FL, USA; Herbert Wertheim College of Medicine, Florida International University, Miami, FL, USA; Department of Neuro-Oncology, Miami Cancer Institute, Baptist Health South Florida, Miami, FL, USA; Herbert Wertheim College of Medicine, Florida International University, Miami, FL, USA; Department of Neurosurgery, Miami Neuroscience Institute, Baptist Health South Florida, Miami, FL 33176, USA; Herbert Wertheim College of Medicine, Florida International University, Miami, FL, USA; Department of Medical Oncology, Miami Cancer Institute, Baptist Health South Florida, Miami, FL, USA; Department of Radiation Oncology, Miami Cancer Institute, Baptist Health South Florida, Miami, FL, USA; Herbert Wertheim College of Medicine, Florida International University, Miami, FL, USA; Department of Radiation Oncology, Miami Cancer Institute, Baptist Health South Florida, Miami, FL, USA; Herbert Wertheim College of Medicine, Florida International University, Miami, FL, USA

**We present the first known case of a multi-recurrent high-grade glioma patient, which after multiple salvage strategies, was treated with a novel radiation therapy technique, “temporally modulated pulsed proton re-irradiation” (TMPPR). This proton therapy (PT) technique has the radiobiological property to allow an enhanced tumor cell death and an increased normal cell sublethal repair by splitting the radiation dose into smaller subfractions delivered as pulses, with the novelty of using PT for its administration. The response to the treatment was impressive, as a complete response (CR) was achieved, minimal treatment-related toxicities were seen, and no deterioration in neurocognitive function was observed.**

Recurrent glioblastoma (GBM) is associated with poor survival. Contemporary salvage treatments have not shown an overall survival (OS) improvement, with minor progression-free survival (PFS) gains in the absence of robust tumor regression. Re-irradiation (reRT) is infrequently used due to concerns of permanent treatment-related damage to the central nervous system, and a recent randomized trial did not demonstrate any OS benefit of adding reRT (35 Gy/10 fractions) to bevacizumab in recurrent GBM.^1^ Even with reRT, significant tumor regression and mass effect resolution are uncommon; CRs remain “unicorns.”^2^

Pulsed reduced dose rate (PRDR) is a reRT technique with temporally interrupted reduced dose pulses, permitting efficient normal tissue DNA repair while enhancing tumor cell kill through a low-dose hyper-radiosensitivity phenomenon.^3^ A single retrospective cohort study showed that photon PRDR was associated with improved survival when combined with bevacizumab over bevacizumab alone, however, CRs were not described.^4^ Photon PRDR is also associated with modest treatment-related toxicities and not widely available.^5^

PT reduces overall integral doses to the brain and critical substructures, of heightened importance in the reRT setting. To harness PT’s dosimetric advantages and the biological benefits of pulsed temporal modulation, we undertook an in-house project to first conduct in-silico dosimetric comparisons,^6^ and then developed, validated, and quality-assured a unique delivery technique (patent pending 63/484,082), which we have designated as TMPPR. We describe, to our knowledge, the first patient treated with this technique.

A 31-year-old woman diagnosed with WHO 2021 CNS grade 4 *IDH1*-mutant (*MGMT* unmethylated astrocytoma, Ki-67 > 80%, 1p19q cointact) in her sixth recurrence presented with intractable seizures, cognitive deficits, and midline shift with early uncal herniation after multiple salvage strategies including four resections (last surgery 2 months prior); systemic therapies (temozolomide [TMZ], CCNU/lomustine and procarbazine, CCNU/lomustine, and vincristine [PCV], one month before); and PT (59.4 Gy/33 fractions at second recurrence, 39 months prior). Salvage bevacizumab plus ivosidenib alone were initially proposed, but after multidisciplinary review, deemed insufficient given her life-threatening situation (young age, aggressive progression, lack of surgical options, and large volume of recurrence [total volume 442.6 cc, enhancing component 105.22 cc]), so TMPPR was added.

TMPPR, 54 Gy/30 fractions, was delivered using pencil beam scanning intensity-modulated proton therapy (PBS-IMPT) with a modified single field optimization approach with 3 fields. Each field was re-painted once, resulting in 6 sub-fields with each delivering approximately 0.3 Gy/fraction. Adjusting for the time delay between each field (5 min) yielded an effective dose rate of 7 cGy/minute, the radiobiological goal for PRDR techniques. The total time for each daily fraction was approximately 37 minutes (positioning and imaging: 10 min, beam on time: 12 min, and wait time between subfraction: 15 min). The patient tolerated treatment well with only CTCAE v5.0 grade 2 alopecia.

**Figure 1.**
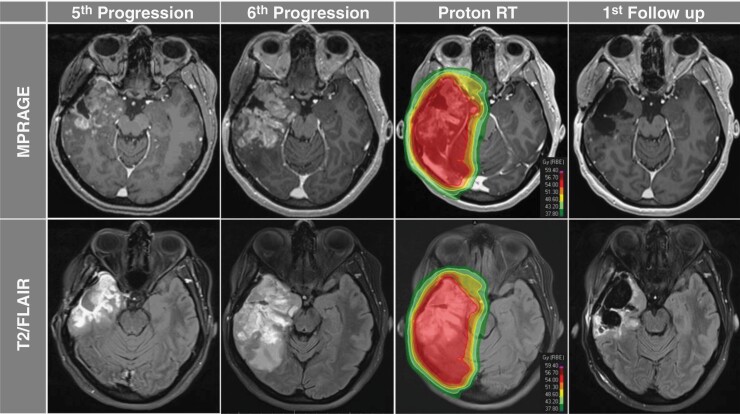
Axial MPRAGE and T2/FLAIR sequences demonstrating the 5^th^ (first column) and 6^th^ (second column) progression after surgery and salvage systemic therapies with significant progression of disease and surrounding mass effect in the right temporal lobe. Temporally modulated pulsed proton re-irradiation (TMPPR) isodose distribution included coverage of the contrast-enhancing tumor and surrounding T2/FLAIR signal (third column). First follow-up MRI performed, 4 weeks after completion of TMPPR demonstrated a complete radiographic response to treatment (fourth column).

Four weeks after TMPPR completion, a brain MRI while on bevacizumab (4 cycles; every 21 days) and ivosidenib (initiated alongside second bevacizumab cycle) and off corticosteroids, revealed dramatic resolution of the large multi-lobulated enhancing mass, substantial reduction in the infiltrating T2/FLAIR signal, resolution of mass effect, and absence of elevated cerebral blood volume (rCBV) on the perfusion MRI (previously increased), consistent with a CR (**Figure 1**).

This case demonstrates successful clinical delivery of TMPPR, validating our planning work. TMPPR was associated with reduced integral doses to the brain compared to photon PRDR, tolerated with minimal toxicity, and resulted in a dramatic complete tumor response with regression of all enhancing and a significant component of the nonenhancing disease as well as resolution of midline shift and mass effect. Such an outcome has not been previously described in multi-recurrent high grade glioma patients treated with photon PRDR, even with bevacizumab, in the published literature, and presents a unique and intriguing case example.

Salvage treatments, even at first recurrence, achieve CR in only 2% of patients.^7^ As this patient was treated with bevacizumab and ivosidenib in addition to TMPPR, the contribution of each, or the combination, to overall outcome cannot be individually discerned. Although bevacizumab is associated with reduction in enhancement, the nonenhancing T2/FLAIR abnormalities characteristically remain stable with no significant reductions in nonenhancing volumes.^8^ Further, preclinical data in the literature suggest that IDH inhibitors delivered concomitantly with radiotherapy can be antagonistic.^9^ Therefore, this patient’s outcome presents an encouraging example of the benefit of reRT, even in a multi-recurrent patient, delivered in this case with TMPPR.

The potential importance of such a dramatic response in recurrent disease was highlighted by Ellingson *et al*. in a review of 4,793 patients over 68 recurrent GBM clinical trials in which a strong correlation between overall response rate and median OS was observed, especially for those with a response rate of greater than 25.^10^ Given the impressive response in this inaugural TMPPR patient, further evaluation of this unique treatment in a prospective clinical trial is warranted.
